# Epstein–Barr Virus (EBV) and Human Papilloma Virus (HPV) in Gastric Cancers, with Special Reference to Gastric Cancer at a Young Age—A Pilot Study in Poland

**DOI:** 10.3390/ijms26020711

**Published:** 2025-01-16

**Authors:** Marek Mazurek, Małgorzata Jaros, Anna M. Gliwa, Monika Z. Sitarz, Ewa Dudzińska, Krzysztof Zinkiewicz, Robert Sitarz

**Affiliations:** 1 Department of Surgical Oncology, Masovian Cancer Hospital, 05-135 Wieliszew, Poland; marekmazurek1@o2.pl; 2 Department of Normal, Clinical and Imaging Anatomy, Medical University of Lublin, 20-950 Lublin, Poland; mjaros090@gmail.com (M.J.); anna.maria.gliwa@gmail.com (A.M.G.); 3 Department of Conservative Dentistry with Endodontics, Medical University of Lublin, 20-090 Lublin, Poland; mksitarz@gmail.com; 4 Department of Dietetics and Nutrition Education, Medical University of Lublin, 20-093 Lublin, Poland; ewa.dudzinska@umlub.pl; 5 Independent Laboratory of Diagnostic, Interventional Endoscopy of the Department of Oncology, Medical University of Lublin, 20-081 Lublin, Poland; kzinek@yahoo.com; 6 Department of Surgical Oncology, St. John’s Cancer Center, 20-090 Lublin, Poland

**Keywords:** gastric cancer, Epstein–Barr virus, human papillomavirus, early-onset gastric cancer

## Abstract

Gastric cancer (GC) is one of the most common cancers in the world. It is a multi-factorial disease influenced by both genetic and environmental factors such as diet, obesity, radiation exposure, and infectious agents. Viral infections usually lead to chronic inflammation, which can initiate the development of cancers. To date, only a few studies have been published about Epstein–Barr virus (EBV) and human papillomavirus (HPV) infections in the context of the development of GC. In particular, research on the development of cancer among people under 45 years of age, including the impacts of EBV and HPV, is rare, and clear results have not been obtained. The aim of this study was to analyze the frequency of occurrence of EBV and HPV in GC, particularly in early-onset gastric cancer (EOGC). Tissue material from 135 patients with GC, including 84 men and 51 women, was examined. RT-PCR was performed to detect EBV, and PCR was performed to detect HPV. There were no significant impacts of EBV and HPV infections on any subtype of GC. There was also no statistically significant dependence of gender and location of the tumor on any subtype of GC. Further research on the impacts of infectious agents such as EBV and HPV on GC should be conducted using larger populations.

## Simple Summary

Gastric cancer (GC) is one of the cancers with the highest morbidity and mortality in the world. Many factors are known that may contribute to the development of this disease. These include infectious agents, among which the Epstein–Barr virus (EBV) and human papillomavirus (HPV) may have potential impacts on the occurrence and course of GC. This study analyzed the prevalence of EBV and HPV in GC, particularly in early-onset gastric cancer (EOGC). Our study confirms previous reports regarding the lack of a significant impact of EBV and HPV infection on the occurrence of all subtypes of GC, as well as highlighting the lack of significant dependence on gender and location of the tumor.

## 1. Introduction

The incidence of gastric cancer (GC) has decreased in many countries. Globally, 1.1 million people were diagnosed with the disease and 770,000 subsequent deaths occurred in 2020. Current statistics indicate that GC is the fifth most common malignancy and the fourth most common cause of death worldwide [1]. The burden of GC has been predicted to increase to ~1.8 million new cases and ~1.3 million deaths by the year 2040 [2].

One of the criteria for dividing GC cases is age at diagnosis. GC can be divided into early-onset gastric cancer (EOGC; 45 years of age or less, without familial genetic predisposition) and conventional GC (over 45 years of age). EOGC is rare (up to about 10% of cases; depending on the publication, the range varies from 2.7% to 15%) [3,4,5,6], and it is more frequent in males than in females [5,7]. It has been suggested that genetic factors are the main reason for its formation, as patients under 45 years of age are less exposed to environmental factors that predispose them to the disease [5,8]. Therefore, EOGC provides an excellent background against which the initiating stages of gastric carcinogenesis may potentially be uncovered.

In addition to the age division, the most popular classification of GC is Lauren’s classification, which takes into account cell morphology and the method of infiltration. Lauren’s classification divides GC into two histological subtypes: intestinal and diffuse. Their different characteristics, including morphology, clinical features, and expansion properties, have consequences for surgical decisions regarding the scope of gastric resection and long-term prognosis. A special type of diffuse subtype is GC with signet ring cells [9].

At present, high-throughput technologies enable comprehensive investigations of GC-related genomic and epigenomic alterations. This improves the ability to identify biomarkers that are significant for the diagnosis of cancers, prediction of outcomes, and creation of individualized treatment regimens. Some of the genetic and epigenetic factors that contribute to the pathogenesis and development of GC include gene mutations, chromosomal abnormalities, differential gene expression, and epigenetic changes. Based on key DNA defects and molecular abnormalities, the Cancer Genome Atlas Consortium (TCGA) divided GC into Epstein–Barr virus (EBV)-positive (8%), microsatellite instability (MSI; 22%), chromosomal instability (CIN; 50%), and genomic stability (GS; 20%) types [10]. This classification was introduced in 2014 and is based on European and US populations.

There are differences in clinicopathological and molecular levels between EOGC and conventional GC. Research has indicated that EOGC is linked to signet ring morphology, Lauren diffuse-type histology, weakly defined histologic grade, Asian/Pacific Islander heritage, and stage IV disease upon diagnosis. EOGC has been linked to both a genomically stable subtype and EBV infection [11]. It has been confirmed that EOGC patients have a higher rate of *CDH1* mutations than and a similar rate of *TP53* mutations to patients with conventional GC [12]. In early-onset diffuse-type GC, significant mutated genes have also been found, such as *BANP*, *MUC5B*, *RHOA*, *ARID1A*, and *TGFBR1* [11].

Of all the classification schemes, the 2010 World Health Organization (WHO) classification is the most comprehensive. It outlines several less-common kinds of stomach tumors, in addition to gastric adenocarcinomas [13]. The four most common histologic types are tubular, papillary, mucinous, and poorly cohesive (including signet ring cell carcinoma) variants. Signet ring cell carcinoma encompasses around 10% of GCs, and it is one of the most difficult to treat [14,15,16].

In 2015, a molecular classification of GC based on next-generation sequencing (NGS) data was established [17], which involved examining the gene expression data of 300 primary gastric tumors. Four molecular subtypes of GC were selected: MSS/TP53(+), MSS/TP53(−), MSI, and MSS/EMT. The above-mentioned classifications of gastric cancer have an impact on clinical activities, as they are associated with different paths of cancer progression, as well as specific treatments and prognoses. Research has indicated that the origin and development of GC may be related to genetic susceptibility factors. Single-nucleotide polymorphisms (SNPs)—a common type of genetic mutation—may accelerate the development of GC. Genome-wide association studies (GWASs) may be able to identify sequence variations in the human genome, screen SNPs related to human diseases, and extend and complement our understanding of associations between genetic variations and cancer risk [18].

GC is a multi-factorial disease, with both genetic and environmental factors contributing to its development [3,19]. Commonly known factors associated with the occurrence of GC include diet (rich in salty, fried, smoked products and preserved foods with nitrogen compounds and poor in fresh vegetables and fruits) [20], smoking, alcohol consumption, obesity [21], and *Helicobacter pylori* (*H. pylori*) infection [22].

Infectious agents are responsible for 50% of cancers (with viruses accounting for 10–15% of cases) [23]. Reduced microbial diversity, altered composition, altered microbial community structure, and aberrant bacterial interactions are all signs of stomach microbiota dysbiosis in GC patients, according to sequencing-based research [24]. The gastrointestinal microbiota has diverse biological functions such as regulating immune responses, preventing the invasion of pathogens, and regulating the central nervous system [25]. Lower diversity and abundance of bacteria have been observed in peritumoral and tumor microhabitats. In addition, the correlation network of interactions in the gastric bacterial community in GC patients was found to be less complex [26]. It has also been confirmed that the presence of *H. pylori* causes changes in the composition of the human stomach microbiota [25]. *H. pylori* infection is the single most common cause of GC, and it has been classified by the WHO as a class I carcinogen since 1994 [22,27]. Studies have shown that *H. pylori* can be cause of chronic inflammation, atrophy, intestinal metaplasia, and low-grade intraepithelial neoplasia, as well as the intestinal type of gastric adenocarcinoma [28,29]. While the risk of infection varies, overall, 15–20% of infected patients develop gastric or duodenal ulcer disease and less than 1% will develop gastric adenocarcinoma [27,30]. The development of GC is caused by *H. pylori* through two primary mechanisms of action: inflammatory processes underlie one of these indirect effects, while the harmful activities of virulence factors directly affect the molecular makeup of gastric epithelial cells in the second pathway [31]. Chronic *H. pylori* infection is the most common cause of gastric mucosa-associated lymphoid tissue (MALT) lymphomas (GMLs). Patients diagnosed with GML, even in the case of remission, have a significantly increased risk of developing gastric adenocarcinoma [32]. *H. pylori* infection is closely associated with the occurrence of primary gastric lymphoma [33]. Moreover, some studies have shown that patients with primary gastric lymphoma are at high risk of developing malignant solid tumors, such as GC [34,35]. GC occurring in individuals of young age has been found to be significantly correlated with the infection, where *H. pylori* was present in 72–82% of cases [36]. It has been established that *H. pylori* contributes to angiogenesis, the immunological microenvironment, and tumor metastasis in GC [31].

In the general population, *H. pylori* and EBV are two known causes of cancer [4,8]. These pathogens can maintain persistent infection in the host body, leading to chronic inflammation that also contributes to the development of cancers. As a result of infection, inflammation and angiogenesis are impaired [37]. The mechanism of action of the virus is that, after the initial infection, it temporarily launches a short lytic program, following which the infection goes into the latency phase [38]. Cancer cells contain the EBV genome and express transforming EBV proteins [5]. EBV microRNAs are often highly expressed, and the over-expression of EBV-miR-BART5-3p has been shown to stimulate the proliferation of GC cells [39,40]. BART5-3p targets the tumor suppressor gene *TP53*, which consequently leads to cell cycle progression and inhibition of cellular apoptosis. It has also been noted that BART5-3p contributes to resistance against chemotherapeutic agents [40]. EBV-related GCs presented significant differences in their phenotypic and clinical attributes, compared to EBV(−) GC. EBV is observed in 7–20% of GCs, and it has been postulated that EBV occurs slightly more frequently in diffuse-type gastric cancers [41,42,43]. The prevalence of EBV(+) GC varies by region of the world [44]. EBV(+) GCs also vary according to patient characteristics, such as sex and age (the incidence decreases with age and among males) [45]. However, there are conflicting studies regarding the survival rates of patients with EBV(+) and EBV(−) GC [31].

As research has shown, coinfection with *H. pylori* and EBV contributes to the development of a highly aggressive form of GC. Both pathogens target host cell polarity complexes. *H. pylori*-associated proteins (CagA, VacAOipA) and EBV-associated genes (LMP1, LMP2A, LMP2B, EBNA1) contribute to the disruption of cellular homeostasis. It has been observed that the maintenance of *H. pylori* virulence is supported by EBV through inactivation of host SHP1 via methylation, thus increasing the carcinogenic nature of the CagA oncoprotein [46]. Among the infectious factors leading to the development of GC, few publications have indicated HPV infection. HPV belongs to the Papillomaviridae family [47]. Many studies have found that HPV infection is a high-risk factor for oncogenesis. HPV types 16, 18, 33, 45, 52, and 58 are associated with various types of cancer [48,49]. Specific systems and organs have been indicated as having the highest risk of developing cancer, including the skin, nasopharynx, genitals, respiratory tract, and digestive tract (esophagus and rectum). HPV oncoproteins disrupt cellular pathways of the cell cycle and apoptosis and stimulate excessive cellular proliferation by exerting an inhibitory effect on p53 proteins [31]. Chronic HPV infection may result in precursor changes such as dysplasia or adenocarcinoma in situ, eventually leading to malignancy [50]. However, the clinical significance of HPV in GC remains unclear and the results of studies in this area are conflicting [31]. Few meta-analyses have focused on HPV infection in gastrointestinal cancers, especially in the Chinese context [51]. HPV is observed in 28% of GC. Additionally, it has been found to be more common in the gastric cardia (36%) and among poorly differentiated GCs (40%) [50]. There are many possible changes and environmental factors that eventually stimulate the pro-carcinogenic activity of genes. Most of these pathways overlap and, therefore, the categorization of carcinogens is very complicated. At present, a key scientific challenge is identifying which infectious factors play a key role in GC, as well as determining the relationships between them and how to prevent their occurrence.

The aim of this study is to analyze the effects of EBV and HPV infection in GC patients, with special reference to the early-onset subtype.

## 2. Results

In 98 samples, DNA of poor quality was obtained, which was considered unsuitable for further stages of the project, not guaranteeing reliable results. For this reason, the study group was limited to 37 cases. Among the 37 patients (28 men and 9 women), 11 were patients with EOGC, while 26 were diagnosed with conventional GC. EBV infection was diagnosed in four patients, and four patients were infected with HPV. Coinfection with HPV and EBV viruses was not observed in any of the patients. A summary of the results obtained is presented in Table 1.

## 3. Discussion

The fourth most common cause of cancer death in the world is GC [2]. EBV is the second pathogen (after *H. pylori*) that may be involved in the development of GC [52]. Only single reports have indicated the impacts of HPV infection on the development of GC [51,53], and many independent reports have confirmed the presence of EBV in GC cells [9,36]. The presence of EBV in GC was first discovered in 1990 using PCR [54] and, at present, EBV is estimated to be present in approximately 10% of GC cases [54,55].

In 2020, Tavakoli et al. conducted a meta-analysis, including 71 scientific reports from 26 countries covering 20,361 patients with GC, and demonstrated the presence of EBV in GC to be 8.77% (95% CI: 7.73–9.92%; I2 = 83.2%). They found that EBV infection is associated with an over 18-fold increase in the risk of GC [54]. In our material, the incidence of EBV in GC was approximately 10% (in the group of patients with conventional GC it was 15.38%), similar to the results of Tavakoli et al. [56]. In 2003, Czopek et al. published the first analysis in Poland on EBV infection and GC development, using a study group consisting of 40 patients. EBV-dependent GC occurred in 12.5% of patients, similar to our results (10.81%) [57]. The researchers concluded that Poland, next to Germany (17.9%), is a country with one of the highest rates of EBV-related GC in the world, unlike Far East countries (e.g., China, Japan), where the percentage ranges from 4.3% to 7% [38,49].

Tavakoli et al. also analyzed the relationship between EBV-related GC and gender. The EBV infection rate was significantly higher among men than among women (10.83% vs. 5.72%; *p* <  0.0001) [56]. The same report analyzed the relationship between EBV infection and Lauren’s classification of GC, in which GC is classified into intestinal and diffuse types. There are many differences between intestinal and diffuse types, based on epidemiology, pathology, and etiology. However, the prevalence of EBV infection was similar between intestinal and diffuse types (*p*  =  0.31) [56]. Other authors have analyzed a Dutch study from 1989 to 1993—called D1D2—which included 566 patients with GC. They found that the prevalence of EBV infection in GC patients was more frequent among men in relation to gender [58]. Other meta-analyses, as presented in Naseem’s summary, have also reported a higher incidence of EBV-related GC among men, with male to female ratios of 2:1 or 3:1 [59,60]. Similar conclusions have been reported by Czopek et al. in a study from 2003, in which EBV-related GC was found in four men and one woman among 40 patients. However, this result was not statistically significant [49]. In our study, EBV infection was found in 14% of men with GC, while the infection did not occur in any of the examined women. However, our result also showed no statistically significant difference between EBV-related GC and gender (*p* = 0.559).

The relationship between EBV and GC among younger patients is controversial. Based on the analysis of the D1D2 study, the occurrence of EBV infection in patients with GC is more common in patients under 60 years of age [58]. Meta-analyses by Murphy et al. from 2009 and Li et al. from 2010 did not show any significant differences with respect to age [60,61]. On the other hand, in 2011, Camargo et al., in a meta-analysis of 5081 patients with GC, found that EBV-related GC was more common among younger patients [45]. In Carvalho’s report from 2004, there was no effect of EBV infection on EOGC. In our study, EBV was not detected in patients under 45 years of age. In the compared groups, there was no statistically significant relationship with EBV infection (*p* = 0.425). Based on Park’s 2016 study, EBV-related GC occurred mainly in the proximal part of the stomach [62]. According to Tavakoli et al., EBV was more prevalent in the cardia (12.47%) and body (11.68%), compared to the pylorus (6.29%) [56]. In the work of Czopek et al., EBV-dependent GC occurred more often in the cardia (two from five patients with positive EBV) and the pylorus (three from five patients with positive EBV) [57]. However, in our results, there was no correlation between the location of the tumor and EBV infection.

The literature indicates that there are heterogeneous cancer cells with different viral loads and variable expression of viral transcripts, but which share common genetic/epigenetic changes [63,64,65]. Lasagna et al. have described this mechanism in terms of a “hit-and-run” theory [65]. It has been shown that, in rare cases, GCs heterogeneously composed of EBV(+) and EBV(−) areas can coexist. Matsuda et al. have described a case of GC with an EBV(+)/TP53(+) component in the peripheral zone surrounded by an EBV(−)/TP53(−) component in the central zone. An explanation for this situation may be a collision tumor of two independent GCs. However, it is also possible that one of the two zones may be metastasis of another primary tumor to the original GC. The authors also pointed out the role of cell-autonomous and non-cell-autonomous mechanisms that may be related to EBV infection [63]. In another case, the authors demonstrated the coexistence of two areas in GC composed of EBV (+)/C-MYC (+) and EBV (−)/HER2 (+) cells, which was described as a collision of EBV(+) and EBV(−) GC, as diagnosed by molecular analysis [64]. These reports may justify the low rate of EBV(+) GCs found in previous studies.

Previous studies have shown the occurrence of HPV in cancers of the cervix, genitals, throat, and some parts of the digestive tract (e.g., oral cavity, esophagus, and anus) [48]. The involvement of HPV infection in GC is still controversial [50,66], and only a few publications have referred to the impact of HPV infection on the development of GC. In 2016, Zeng et al. published a large meta-analysis including 30 studies (1917 GC patients and 576 control patients) and concluded that HPV plays a potential role in the pathogenesis of GC. The prevalence of HPV was 28.0% among patients with GC. The prevalence of HPV was significantly higher among Chinese patients than patients from non-Chinese regions of the world (31% vs. 9%) [50], and HPV was detected in 44.4% of male patients and 29.7% of female patients with GC. In our study, there was no statistically significant relationship between gender and HPV infection (*p* = 0.515). The prevalence of HPV was higher in those with gastric cardia cancer than general GC (36% vs. 28%) [27]. In our study, in the group with cardia cancer, 20% of patients had HPV infection, but there was no statistically significant relationship between the location of the cancer and HPV infection (*p* = 0.617).

In 2020 Wang et al. selected 14 studies assessing the impacts of HPV infection on the development of GC (901 patients and 1205 controls). A meta-analysis confirmed the relationship between the incidence of HPV and GC. The overall prevalence of HPV in GC was 23.6% (*p* < 0.001) [67]. Bae summarized both of the above studies [51]. In Zeng’s analysis, 12 papers concerned Chinese research while, in Wang’s research, it was 5 papers. There were many differences between their meta-analyses, and it is worth noting the statistically significant difference in the pooled OR and their 95% CIs (Zeng 3.88–14.1, Wang 1.53). Chinese studies have shown that HPV infections increase the risk of GC, unlike studies from outside China (there was no statistical significance, similar to our study).

In 2014, Snietura et al. analyzed 84 patients with respect to the impact of HPV infection on the development of GC in Polish patients. DNA of HPV was not found in any of the tissue samples taken from patients with GC during surgical procedures [68]. Similar results were published by Roesch-Dietlen et al. in 2018, in a paper presenting the cases of 53 patients with gastrointestinal cancer. GC was diagnosed in 10 patients, while HPV was not detected in any of them [69]. According to Polish data from 2020, HPV-related GC occurred in 21.9% of men and 12.1% of women with GC [70]. These results are contrary to those of our research, in which 7% of men and 22% of women were diagnosed with HPV-related GC.

In Zeng’s study, 75 of 223 patients with GC aged > 50 years were HPV-positive; meanwhile, in those aged < 50 years, HPV was observed in 45 of 106 patients. There was no significant difference between the prevalence of HPV infection and the age of patients with GC [27]. According to Polish statistics, among patients with HPV-related GC under 45 years of age, 1.4% are men and 1.61% are women [70]. In our study, only one patient under the age of 45 was diagnosed with HPV, which turned out to be statistically insignificant. In 2018, de Souza et al. conducted research on the correlation of HPV, EBV, and *H. pylori* coinfection with GC [71]. In the 302 samples tested, all three pathogens were found, including *H. pylori* in 87%, EBV in 20%, and HPV in 3%. Based on the results, it was concluded that HPV does not play a role in the development of GC. Moreover, in the study, the fundus of the stomach was the region least affected by pathogens, which is contrary to Zeng’s studies [50,51].

## 4. Material and Methods

### 4.1. Material

The material, in the form of 135 tissue paraffin blocks, was taken during endoscopy of the upper gastrointestinal tract and during surgery from patients (84 men and 51 women) with GC before chemotherapy or radio-chemotherapy. The material was divided into two groups. The first group consisted of patients up to 45 years of age (EOGC, 34 patients), while the second group included patients over 45 years of age (conventional GC, 101 patients). The anatomical location of GC was determined according to the ICD10 classification (the 10th revision of the International Classification of Diseases). Detailed characteristics of the study group are presented in Table 2.

The material came from patients diagnosed and treated in the 2nd Department of General Surgery, Gastroenterology and Digestive Tract Cancers of the Independent Public Clinical Hospital No. 1 in Lublin and the Department of General and Oncological Surgery of the John Paul II Hospital in Zamość. This was a retrospective study.

The research project received consent from the Bioethics Committee (KE-0254/139/2019) at the Medical University of Lublin.

### 4.2. Methods

Before the DNA isolation stage, the paraffin sections with tissue were deparaffinized. DNA isolation was performed using the QIAamp DNA Mini Kit (Qiagen, Hilden, Germany), according to the manufacturer’s instructions and our own experience [72]. To verify the quality of the obtained DNA and the presence of PCR inhibitors, the reaction for human β-globin was performed using primers PC04 and KM29. The sequence of the set primer of PC04 was d(5′-CAA CTT CAT CCA CGT TCA CC-3′), while that for KM29 was d(5′-GGT TGG CCA ATC TAC TCC CAG G-3′) (product 205 pb) [68]. After analyzing the material using the reaction for human β-globin, it was found that only 37 samples could be used for the next stage of research.

Detection of the HPV genetic material was performed via PCR using the specific primers MY09 and MY11. The sequence of the set primer of MY09 was d(5′ CGTCCMARRGGAWACTGATC 3′), and that for MY11 was d(5′ GCMCAGGGWCATAAYAATGG 3′) (product 450 pb) [73].

Detection of EBV genetic material in isolates was performed via RT-PCR. The real-time PCR test was performed using the ready-made Epstein-Barr Virus PCR Kit (GeneProof, Brno, Czech Republic) test, according to the manufacturer’s instructions [74].

Due to the fact that the number of samples tested was not large, the tests were repeated in 10% of cases and the results were confirmed. The relationship between the analyzed qualitative variables was assessed using the chi^2^ test and the odds ratio test. The level of *p* < 0.05 was taken to indicate statistical significance.

## 5. Conclusions

In our research, the incidence of HPV infection in GC was 10.81%, which is similar to that in previous reports. Furthermore, the results confirmed the incidence of EBV infection in GC at 10.81%, also similar to previous reports. Studies conducted to date have indicated the impacts of EBV and HPV infections on the occurrence and development of GC. However, in this study, there were no significant relationships between the occurrence of EBV or HPV infection and the GC subtype, gender, and tumor location. In summary, there is a need to conduct further research regarding the impacts of infectious agents on the development of GC, with particular emphasis on EOGC in larger populations. Our results may be subject to error due to the small number of cases, although similar results have previously been published.

## Figures and Tables

**Table 1:** Summary of the results.

Analyzed Variable			Group		Chi^2^ *p*	OR, 95% CI, *p*
			Up to 45 Years	Over 45 Years		
Age	M ± SD		40.82 ± 4.31	68.65 ± 9.15	–	–
	Me [Q1–Q3]		43 [40–44]	69.5 [65–75]		
	Min–Max		30–45	47–82		
Sex	Men	N	7	21	Chi^2^ = 0.478 *p* = 0.490	OR = 0.417 95% CI: 0.087–2.000 *p* = 0.274
		%	63.64	80.77		
	Women	N	4	5		
		%	36.36	19.23		
Diagnosis	Other than C.16	N	6	21	Chi^2^ = 1.530 *p* = 0.216	OR = 3.5 95% CI: 0.753–16.263 *p* = 0.110
		%	54.55	80.77		
	C16.0: cardia	N	5	5		
		%	45.45	19.23		
EBV	Negative	N	11	22	Chi^2^ = 0.637 *p* = 0.425	OR < 0.001 *p* = 0.999
		%	100.00	84.62		
	Positive	N	0	4		
		%	0.00	15.38		
HPV	Negative	N	10	23	Chi^2^ = 0.130 *p* = 0.719	OR = 0.767 95% CI: 0.071–8.299 *p* = 0.827
		%	90.91	88.46		
	Positive	N	1	3		
		%	9.09	11.54		
All		N	11	26	–	

Abbreviations: M ± SD, geometric mean plus or minus standard deviation; OR, odds ratio; CI, confidence interval; Chi^2^, statistical test; *p*, significance; diagnosis, gastric cancer anatomical location according to the ICD10 classification (the International Classification of Diseases).

**Table 2:** Characteristics of the research group.

Analyzed Variable		N	%
Age	Up to 45	34	25.19
	Over 45	101	74.81
	M ± SD	61.98 ± 15.48	
	Me [Q1–Q3]	66 [45–73]	
	Min.–Max.	26–85	
Sex	Men	84	62.22
	Women	51	37.78
Diagnosis	C16.0: cardia	20	14.81
	C16.1: fundus	6	4.44
	C16.2: body	36	26.67
	C16.3: pyloric antrum	9	6.67
	C16.4: pylorus	9	6.67
	C16.5: less curvature	3	2.22
	C16.6: greater curvature	2	1.48
	C16.8: lesion extending beyond the boundaries of one site within the stomach	21	15.56
	C16.9: stomach, unspecified location	29	21.48
All		135	100.00

Abbreviations: M ± SD, geometric mean plus or minus standard deviation; diagnosis, gastric cancer anatomical location according to the ICD10 classification (the International Classification of Diseases).

## Data Availability

The data generated and analyzed during the current study are available from the corresponding author upon reasonable request.
